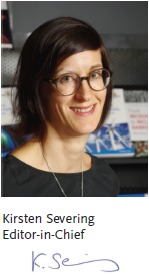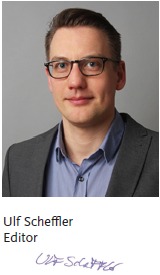# The Success Story Continues …

**DOI:** 10.1002/advs.201600494

**Published:** 2017-01-16

**Authors:** Ulf Scheffler, Kirsten Severing

**Figure 1:**
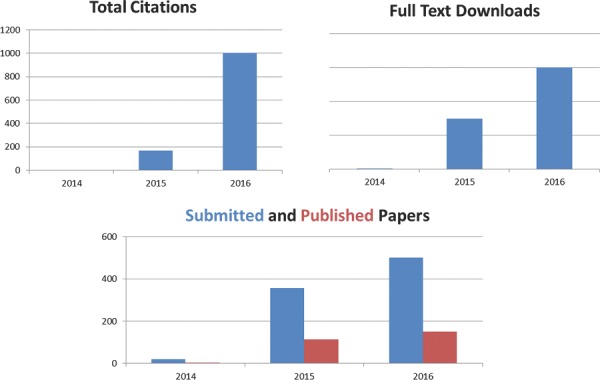
Key performance indicators for *Advanced Science* during the first years.

Last year at this time, we had many reasons to celebrate. *Advanced Science* had completed its first whole year with the publication of its 12^th^ issue. Shortly before Christmas we had been informed that *Advanced Science* had been accepted for coverage in *Web of Knowledge* – an obvious milestone in the history of our young journal. It took another three months before the content was actually to be found in *Web of Knowledge*. Afterwards, the discoverability of the journal rose significantly, leading to a strong increase in the number of full text downloads as well as citations of articles published in the journal. In June, we had the pleasure to celebrate our first ISI Impact Factor of 6.00. Some key performance indicators can be seen in **Figure** 1 that all show that the journal is still on the rise.


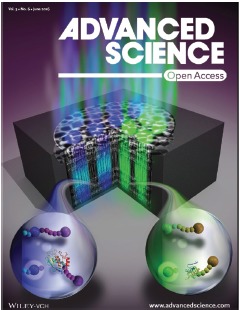


While the number of full text downloads has doubled, the number of citations has increased by a factor of six (at the time of writing) as compared to the previous year. The number of submissions has also grown by approximately 40%. The high quality of the submitted papers as well as the broad topical range of the manuscripts suggests that the journal has already established the reputation of a premium publication platform for fundamental and applied research in materials science, physics and chemistry, medical and life sciences, as well as engineering. The immediacy index at the time of writing is around 2 compared to 1.5 in 2015. The expectations are therefore high that also the impact factor will increase significantly next year.


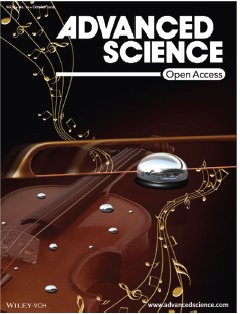


**Table** 1 presents the highest cited papers in 2016 (published in 2014 and 2015) which will be the main driving force for the next ISI impact factor. Popular topics include solar cells, batteries, but also photocatalysts, metal‐organic frameworks, and hydrogel composites. Since the journal was included in *PubMedCentral* the journal has also received more and more biomedical and life sciences related contributions. Among the most read articles (http://onlinelibrary.wiley.com/journal/10.1002/(ISSN)2198‐3844/homepage/2749_mostaccessed.html) you also find papers on tumor markers and cardiac tissue repair.

**Table 1:** Highest cited papers in 2016

Title	Corresponding Author	Publication Date	Total Citations	Citations in 2016
Control of Emission Color of High Quantum Yield CH_3_NH_3_PbBr_3_ Perovskite Quantum Dots by Precipitation Temperature	Andrey L. Rogach et al., *City University Hong Kong*, China	September 2015	43	39
The Rational Design of a Single‐Component Photocatalyst for Gas‐Phase CO_2_ Reduction Using Both UV and Visible Light	Geoffrey A. Ozin et al., *University Toronto*, Canada	December 2014	28	23
Pure Single‐Crystalline Na_1.1_V_3_O_7.9_ Nanobelts as Superior Cathode Materials for Rechargeable Sodium‐Ion Batteries	Xin‐Bo Zhang et al., *Chinese Academy of Science*, Changchun, China	March 2015	23	20
Nanoparticle‐Hydrogel Composites: Concept, Design, and Applications of These Promising, Multi‐Functional Materials	Xian Jun Loh et al., *National University Singapore*, Singapore	February 2015	29	19
Electrode Nanostructures in Lithium‐Based Batteries	Yanglong Hou et al., *Peking University*, China	December 2014	27	18
Porous Nickel‐Iron Oxide as a Highly Efficient Electrocatalyst for Oxygen Evolution Reaction	Rui Cao et al., *Shaanxi Normal University*, Xian, China	October 2015	18	17
Recent Progress in Electronic Skin	Caofeng Pan et al., *Chinese Academy of Science*, Beijing, China	October 2015	18	17
A Tetraperylene Diimides Based 3D Nonfullerene Acceptor for Efficient Organic Photovoltaics	Alex K.‐Y. Jen et al., *University of Washington*, USA	April 2015	23	16
Growth of Ultrathin ZnCo_2_O_4_ Nanosheets on Reduced Graphene Oxide with Enhanced Lithium Storage Properties	David Lou et al., *Nanyang Technology University*, Singapore	February 2015	23	16
Construction of Efficient 3D Gas Evolution Electrocatalyst for Hydrogen Evolution: Porous FeP Nanowire Arrays on Graphene Sheets	Xin Wang et al., *Nanyang Technology University*, Singapore	August 2015	18	16
Pure Single‐Crystalline Na_1.1_V_3_O_7.9_ Nanobelts as Superior Cathode Materials for Rechargeable Sodium‐Ion Batteries	Xin‐Bo Zhang et al., *Chinese Academy of Science*, Changchun, China	March 2015	18	16
High‐Volume Processed, ITO‐Free Supstrates and Substrates for Roll‐to‐Roll Development of Organic Electronics	Frederik C. Krebs et al., *Technical University Denmark*, Roskilde, Denmark	December 2014	23	13

Our contributions originate from various countries, reflecting regional diversity and covering the full scope of modern science. 45% of our articles are provided by Asian authors, mainly from China, Singapore and Japan. This is followed by European (21%), American (17%), and Australian (6%) manuscripts. Accordingly, a wide international readership is reached, especially, since all research articles published in *Advanced Science* are immediately freely available to read, download and share. This accessibility is increased further by the introduction of the new *Advanced Science App* (https://itunes.apple.com/us/app/advanced‐science/id1070856891?ls=1&mt=8).

We would like to thank all editorial board members, authors, and reviewers for their ongoing support and their indispensable contributions to the success story of this young journal. Furthermore, we are looking forward to many years of fascinating research.

On behalf of the editorial team,